# Genetic Diversity Underlying the Envelope Glycoproteins of Hepatitis C Virus: Structural and Functional Consequences and the Implications for Vaccine Design

**DOI:** 10.3390/v7072809

**Published:** 2015-07-17

**Authors:** Alexander W. Tarr, Tanvi Khera, Kathrin Hueging, Julie Sheldon, Eike Steinmann, Thomas Pietschmann, Richard J. P. Brown

**Affiliations:** 1 School of Life Sciences, Nottingham Digestive Diseases Biomedical Research Unit, University of Nottingham, Nottingham NG7 2RD, UK; 2 Institute of Experimental Virology, TWINCORE, Centre for Experimental and Clinical Infection Research, A Joint Venture between the Medical School Hannover (MHH) and the Helmholtz Centre for Infection Research (HZI), Hannover D-30625, Germany; E-Mails: tanvi.khera@twincore.de (T.K.); kathrin.hueging@twincore.de (K.H.); julie.sheldon@twincore.de (J.S.); eike.steinmann@twincore.de (E.S.); thomas.pietschmann@twincore.de (T.P.); 3 German Centre for Infection Research (DZIF), partner site Hannover-Braunschweig, Braunschweig 38124, Germany

**Keywords:** HCV, glycoproteins, structure, evolution, diversity, antigenicity, functionality, vaccine

## Abstract

In the 26 years since the discovery of Hepatitis C virus (HCV) a major global research effort has illuminated many aspects of the viral life cycle, facilitating the development of targeted antivirals. Recently, effective direct-acting antiviral (DAA) regimens with >90% cure rates have become available for treatment of chronic HCV infection in developed nations, representing a significant advance towards global eradication. However, the high cost of these treatments results in highly restricted access in developing nations, where the disease burden is greatest. Additionally, the largely asymptomatic nature of infection facilitates continued transmission in at risk groups and resource constrained settings due to limited surveillance. Consequently a prophylactic vaccine is much needed. The HCV envelope glycoproteins E1 and E2 are located on the surface of viral lipid envelope, facilitate viral entry and are the targets for host immunity, in addition to other functions. Unfortunately, the extreme global genetic and antigenic diversity exhibited by the HCV glycoproteins represents a significant obstacle to vaccine development. Here we review current knowledge of HCV envelope protein structure, integrating knowledge of genetic, antigenic and functional diversity to inform rational immunogen design.

## 1. Introduction

Discovered as the causative agent of non-A non-B hepatitis in 1989 [1], the hepatitis C virus (HCV) is a member of the genus Hepacivirus. While the origins of HCV in humans remains undefined, multiple recent discoveries of related hepaciviral homologs infecting diverse mammalian species have been reported (reviewed in [2]). However, the species and tissue tropism of HCV is highly restricted with the virus only naturally infecting human hepatocytes, although chimpanzees can be experimentally infected [3]. HCV represents an important human pathogen, causing a significant disease burden and associated healthcare costs worldwide. Indeed, the virus is disseminated globally, infecting 2%–3% of humanity [4,5]. A chronic infection ensues in around 80% of cases, which is often asymptomatic, predisposing carriers to the risk of potentially fatal liver disease which increases with duration of infection. HCV is an enveloped virus and possesses a 9.6 kb + sense RNA genome encoding a single open-reading frame, which is translated as a single polyprotein. Co- and post-translational cleavage by host and viral proteases into 10 mature viral proteins then occurs. The non-structural proteins are involved in viral RNA replication, contribute to virion assembly but are not incorporated into virions. The structural proteins include the viral capsid encoded by the *core* coding-region and the envelope glycoproteins E1 and E2 encoded by the *E1* and *E2* coding-regions. The glycoproteins reside on the surface of virion and represent complex multifunctional proteins, which perform an array of biological functions. As the name suggests, they are heavily glycosylated with complex sugar moieties and represent the key determinants for HCV entry into permissive cells, facilitating receptor binding and mediating the fusion process between viral envelope and endosomal host cell membrane. The proteins also contribute to virion assembly, bind host lipoproteins and interfere with host innate immune responses. While these critical functions must be maintained, HCV glycoproteins are the targets of host adaptive immune surveillance and must evolve to escape detection, which is facilitated by the error-prone nature of viral replication. In this review we summarize current knowledge of all aspects of HCV glycoprotein biology, including structure, genetic diversity, antigenicity and functionality, in addition to the current status of potential vaccination strategies.

## 2. Structure of the HCV envelope glycoproteins

The HCV glycoproteins are incorporated into the viral lipid envelope during budding of virus particles [6]. By comparison to related members of the *Flaviviridae* family these proteins are thought to possess all the necessary receptor binding determinants and fusion peptide required for cellular entry in a clathrin-dependent, endosomal pathway [7,8,9]. Indeed, these glycoproteins are both necessary and sufficient for mediating the HCV entry cascade of all strains of HCV [10,11,12]. Both E1 and E2 are essential for infectivity [10], with E2 possessing the binding sites for the entry receptors CD81 [13] and SCARB1 [14]. Expression of these proteins in *cis* enhances protein integrity [15] and virus infectivity [10], but heterodimerization does occur when the two proteins are expressed in *trans* [16]. It has been possible to make trans-complemented infectious virus particles using pseudoviruses [17] or sub-genomic packaging replicons to make HCV-transcomplemented particles (TCP) [18,19,20,21,22]. Heterodimers are believed to occur through interactions in both the transmembrane domains [23,24,25,26] and the ectodomains [17,27] of E1 and E2. Both proteins possess unusually extensive glycosylation, which despite substantial genetic variability, is highly conserved between strains [28,29,30]. This carbohydrate component makes up approximately half of the mass of the two glycoproteins, and has a role in protein folding and correct expression of the glycoproteins [28,29]. There is also extensive disulphide bridging in both proteins. The 18 cysteine residues in E2 are conserved and necessary for production of infectious viruses [31]. This is also the case for the 8 conserved cysteines in E1, although there appears to be low levels of function in alanine-replacement mutants at these sites [32]. As well as their essential role in entry, these proteins mediate steps in the virus assembly process prior to capsid envelopment [33] and must be expressed together to produce correctly-folded proteins [34] and infectious virus particles [35,36].

Early evidence suggested that the structure of the E2 protein was analogous to the Class II fusion proteins found in all other flaviviruses [37], while E1 was also predicted to have regions functionally homologous to that of Tick-Borne Encephalitits virus (TBEV) fusion protein [38]. Structural modelling of the HCV E2 protein based on the TBE glycoprotein E sequence revealed differences between the two viruses, but highlighted global similarities in the amino acid sequences that supported the hypothesis of similar structures between these two viruses [37]. A dimer of HCV E2 protein on the surface of infectious virus particles was predicted, as seen in dengue virus [39], with a trimeric post-fusion conformation [40]. E1 has been described in a homotrimeric complex [6,32]. The proposed flavivirus-like E2 architecture was also supported by analysis of the conserved intramolecular disulphide-bridging patterns [41], although more recent evidence has suggested alternative disulphide patterns also exist [42]. The fusion pathway is yet to be resolved, but both E1 and E2 have proposed fusion peptides [43,44,45,46], indicating that both proteins may contribute to fusion in a non-conventional fusion pathway.

**Figure 1:**
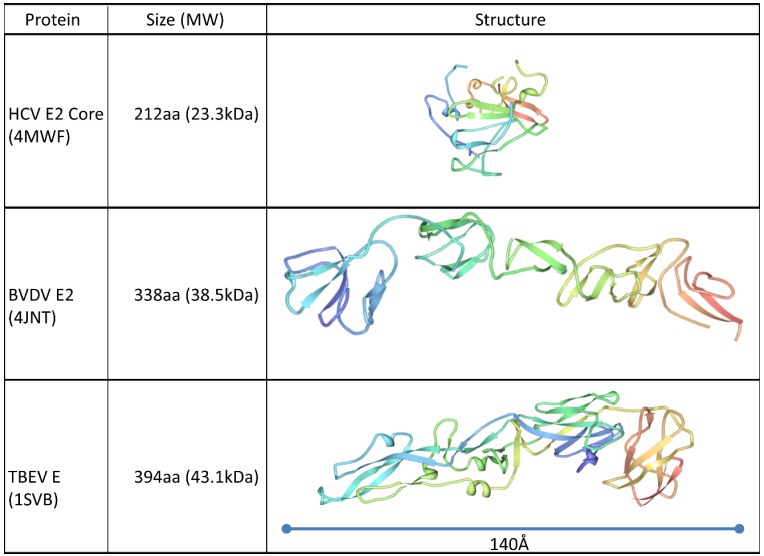
Comparison of the structures of the HCV E2 glycoprotein core [42] with the crystal structures of Bovine Viral Diarrhoea Virus E2 glycoprotein [48] and Tick-Borne Encephalitis virus E glycoprotein [67]. The resolved crystal structure of HCV E2 revealed a much more compact protein structure than that of other members of the family Flaviviridae. Protein ribbons are coloured from the N-terminus (blue) through to the C-terminus (red). The PDB ID numbers are indicated in brackets.

**Figure 2:**
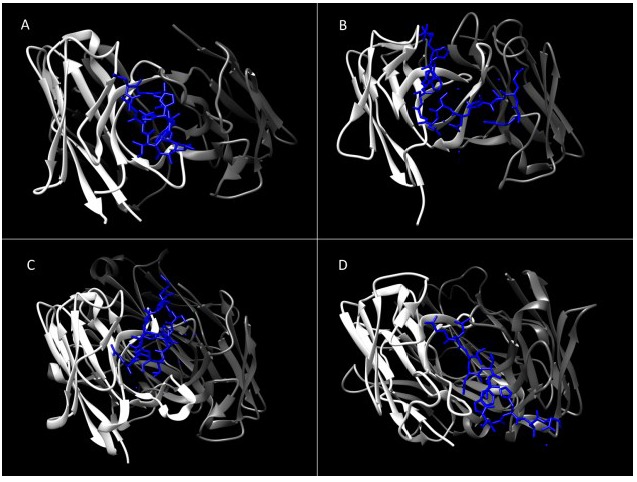
Structural flexibility in the N-terminus of E2. Peptides representing the amino acids 412–424 of the HCV polyprotein (blue) were crystalized in complex with monoclonal antibodies (in each case the heavy chain is highlighted in white and light chain in grey). (**A**) AP33 [63,65]; (**B**) HC33.1 [66]; (**C**) HCV1 [65]; (**D**) 3/11 [62]. The contrasting structures of this region when in complex with different antibodies, ranging from a β-turn structure (**A**,**C**) to an extended coil (**B**,**D**). This suggests that the conserved N-terminus of E2 can adopt an array of alternative conformations on the surface of the protein. This inherent flexibility could be a mechanism to evade neutralizing antibody responses, or could highlight the different conformations adopted at different stages of the entry cascade.

The possibility of alternative structural organization of HCV E1 and E2 compared to Class II fusion proteins was highlighted by the discovery of a completely novel class of fusion protein for the genetically related Bovine Viral Diarrhoea Virus (BVDV) E2 glycoprotein [47,48]. More recent crystallographic analysis of the conserved core of the HCV E2 protein has revealed a protein fold dissimilar to the glycoproteins of all other members of the Flaviviridae family, with a compact Ig-like domain surrounded by flexible variable regions on the antibody-accessible surface of the protein [42,49]. This protein shares little structural homology to any other described fusion proteins (See Figure 1). This difference in structure compared to the Class II viral fusion proteins is consistent with the lack of a regular glycoprotein network on the surface of HCV virions, contrasting with other flaviviruses [50,51]. The E2 core possesses three distinct surface features: A highly glycosylated face; an occluded face, and a conserved face possessing epitopes of broadly neutralizing antibodies [42]. This neutralization face possesses the conserved residues involved in the interaction with CD81. Three discontinuous regions are known to contribute to this face, originally identified as targets of neutralizing antibodies recognizing linear epitopes around amino acids 412–423, 436–446 and 523–538. Site-directed mutagenesis indicated that amino acids necessary for the interaction with CD81 were located at W420 in Region 1, W437, L438, L441, and F442 in Region 2, and Y527, W529, G530 and D535 in Region 3 [52,53,54]. On the resolved crystal structure of E2, these discontinuous residues were revealed to be exposed, co-located on a receptor binding face that included the “front layer” (Region 2) and a bilobular loop representing Region 3 [42]. However, in these structural studies the hypervariable region 1 (HVR1), which interacts with both apolipoprotein E (ApoE) and SCARB1 [55,56,57,58,59] was deleted, preventing resolution of this receptor binding site. The highly conserved face of E2 possessing the CD81 binding determinants is a target for neutralizing antibodies, with the genetically conserved N-terminal region downstream of the HVR1 possessing key immunogenic epitopes [60,61] Intriguingly, this conserved region was found to be disordered in the resolved structure, suggesting that this is a flexible region that does not adopt a regular conformation. This is in contrast to its highly conserved sequence and essential role in CD81 binding and entry [62]. Independent structural studies have confirmed this conformational flexibility, capturing the conserved N-terminus in a variety of discrete conformations when complexed with different monoclonal antibodies [62,63,64,65,66]. This highlights a potential novel mechanism that has evolved to allow HCV to evade neutralizing antibodies, consistent with the ability of this region to elicit different antibody specificities during chronic infections while maintaining identical primary amino acid sequences [60,61] (see Figure 2).

While the crystal structure of E2 core has enhanced our understanding of the features of E2, detailed information on the structure of E1 is still lacking. Full length E1 is difficult to express alone [68,69,70], limiting studies on purified protein. However, a recently-described structure of the N-terminal 79 amino acids of E1 has demonstrated that this protein forms a covalently linked dimer with structure similar to the phosphtidylcholine transfer protein [71]. It is yet unclear if this structure corresponds to the structure on the virion, a post-attachment structure, or a post-fusion fold. However, these structural data provide new insights into the antigenic structure of E1 and E2.

## 3. Genetic Diversity in HCV E1 and E2

Continued international sampling efforts have resulted in a rapid growth in viral sequences deposited in international sequence databases, with HCV second only to HIV-1 in terms of numbers of sequences [72]. This expansion is driven by increasing clinical significance with HCV responsible for 700,000 deaths worldwide in 2013 [73]. Analysis of sequences deposited reveals HCV exhibits extreme genetic heterogeneity worldwide. This diversity is the product of the low fidelity of the viral RNA dependent RNA polymerase (RdRp) coupled with the high replication rate *in vivo*. The HCV *E1E2*-coding region exhibits extreme variation. This underlying genetic variability occurs despite the requirement for essential interactions between the encoded glycoproteins and lipoproteins [33,74] and cellular receptors [13,14]. Indeed, conserved receptor interactions required for the HCV entry cascade have been mapped to highly conserved residues in the E2 protein [52,53,54,75,76]. The evolution of these coding-regions is influenced by dynamic host-virus interactions, with an ever changing adaptive immune response shaping the diversity of the intrahost viral population. Consistent with their role in the conserved entry pathway of HCV into hepatocytes, the envelope glycoproteins have many highly conserved amino acid sites [77]. Most of these conserved regions in E2 make up the conserved core of the protein presented in the crystal structure. The less well-structured regions generally display more genetic diversity, particularly the three variable regions: HVR1, HVR2 and the intergenotypic variable region (IgVR). Unfortunately clinical HCV isolates cannot be readily propagated in tissue-culture, limiting our understanding of the functional traits of diverse HCV glycoproteins. Indeed, only a handful of genotype 2-based viruses are able to effectively replicate [35,78] and thus the functional correlates of this vast global sequence diversity are incompletely defined.

### 3.1. Global Diversity

HCV exhibits substantial genetic diversity worldwide with classification of diversity requiring periodic updating to keep pace with constant influx of deposited sequences [79,80]. Phylogenetic analysis reveals current circulating HCV strains cluster into seven divergent genotypes, which can be further subdivided into 67 subtypes [80]. In addition to regional variations in genotype and subtype prevalence, different routes of transmission and differential rates of spread within and between diverse at-risk populations occurs [4,5,80,81,82,83,84,85]. The global HCV phylogeny is characterized by long internal branch lengths between genotypes and subtypes [80,86]. This diversity surpasses the diversity apparent in the group M HIV-1 pandemic due to an increased period of virus-host association [86]. Based on genetic diversity, a clear distinction can be made between members of the same subtype (<13% nucleotide difference over the entire coding region) and different subtypes or genotypes (>15% nucleotide difference) [80]. Over the entire HCV genome individual genotypes differ at between 30%–35% of nucleotide sites, with 20%–25% nucleotide variability apparent between subtypes [87].

However, this genomic heterogeneity is not uniformly distributed but instead concentrated in regional hot-spots. The *E1* and *E2* coding-regions represent one of the most divergent regions of the HCV genome, with particularly high variation apparent in the HVR1 [87]. HVR1 displays greater than 60% genetic diversity between functional isolates, greater than any other part of the HCV genome. Within HVR1, positive selection (*d*_N_/*d*_S_ >1) is observed at subset of homologous sites, irrespective of infecting genotype, with a genotype-specific distribution of positively selected sites observed in the remainder of *E2* [77]. Despite this, the majority of codons in *E1E2* are under strong negative selection (*d*_N_/*d*_S_ <1), with most substitutions in codon triplets occurring at silent sites implying strong structural/functional constraints place limits on E1E2 diversity [77]. Indeed, divergent restrictions on fixation of non-synonymous mutations in globally sampled glycoprotein sequences limit the mutational space which can be effectively explored by RNA viruses, with selectively deleterious mutations in E1E2 reducing either virion assembly, target cell entry or enhancing host antibody recognition and thus limiting their fixation in globally sampled strains [88].

**Figure 3:**
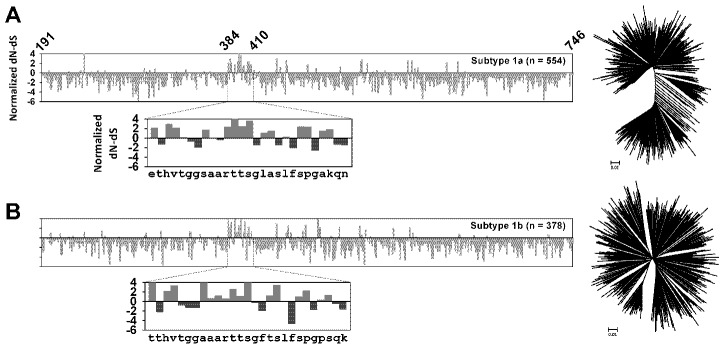
Characterization of positive and negative selection in HCV subtype 1a and 1b envelope glycoproteins. For each panel, full-length E1E2 nucleotide sequences were downloaded from GenBank and aligned according to encoded amino acid sequence. A comparative analysis of selection acting on contemporaneously sampled subtype 1a (**A**) and 1b (**B**) glycoprotein sequences is presented. For both data sets, normalized dN-dS values across the E1E2 coding-region are presented, with the test statistic obtained using the total number of substitutions in the tree (measured in expected substitutions per site). The test statistic dN-dS is used for detecting codons that have undergone positive selection, where dS is the number of synonymous substitutions per site (s/S) and dN is the number of nonsynonymous substitutions per site (n/N). Positive values indicate an overabundance of non-synonymous substitutions and are indicative of adaptive evolution. Negative values are indicate an overabundance of synonymous substitutions and are indicative of purifying selection due to structural/functional constraint. The numbers of sequences analysed for each genotype are presented in the top right corner of each plot. A magnified plot of selection in HVR1 (E2 residues 384–410) is positioned below each full-length E1E2 selection plot, with positively selected sites highlighted in red and negatively selected sites highlighted in blue. Subtype 1a and subtype 1b HVR1 consensus amino acids are located below each HVR1 plot. Phylogenetic trees representing the sequences utilized for each analysis are positioned to the right of each E1E2/HVR1 plot. Branch lengths are equivalent to genetic distance measured in nucleotide substitutions per site and are proportional to the scale bar. Maximum Likelihood computations of dN and dS were conducted using HyPhy [97] and MEGA6 [98].

**Figure 4:**
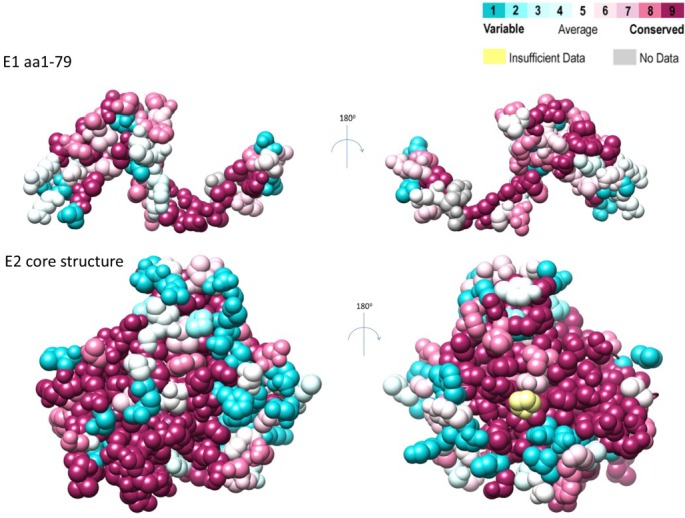
Conservation and variability in the E1 and E2 proteins. Conservation plots were generated using an alignment of 866 genotype 1 full-length E1E2 amino acid sequences retrieved from GenBank. Alignments were plotted using the ConSurf server [99], highlighting the variability in the first 79 amino acids of E1 (mapped onto pdb 4UOI), and the core structure of E2 (mapped onto pdb 4MWF). Conserved amino acids are represented in mauve, and highly variable amino acids in cyan. This analysis revealed a mainly conserved exposed face of the N-terminus of E1, with some variable residues buried in the monomer of the N-terminus of E1. It also showed two conserved faces of the core E2 structure (highlighted by contiguous mauve residues in the two diagrams), with a variable discontinuous region (highlighted with patches of cyan residues) surrounding the conserved CD81 binding residues and neutralizing epitopes.

Genotype 1 viruses infect 84 million people worldwide and represent the predominant circulating strain in developed nations [81,82]. Consequently the majority of sequences deposited in databases are derived from genotype 1 strains. To assess the evolutionary pressures which shape current global genotype 1 diversity, a comparative *d*_N_/*d*_S_ analysis, scanning the entire *E1E2* coding-region and incorporating representative sequences from subtype 1a and 1b can be seen in Figure 3. Sites undergoing adaptive evolution (normalized *d*_N_-*d*_S_: Positive values) are detected throughout the *E1E2* coding-region, focused in HVR1 and similar (although not identical) between subtypes 1a and 1b. However, purifying selection (normalized *d*_N_-*d*_S_: Negative values) dominates the evolution of this coding-region. The detection of positive selection at a subset of *E1E2* codons in subtype 1a and 1b viruses likely represents the footprints of viral escape from host immune targeting (Figure 3). These data also reveal *E1E2* regions restricted in their ability to tolerate mutations, in conjunction with more mutationally flexible regions. Additionally, for the first time, the conservation and variability apparent in circulating genotype 1 strains (subtype 1a and 1b combined) was mapped onto both the recently described E1 N-terminus (upper panels) and the E2 core structure (lower panels) and is presented in Figure 4. Together these analyses, which incorporate freely available data downloaded from public databases, can guide future immunogen design strategies (Figure 3 and Figure 4). Indeed, HCV vaccination approaches should elicit maximum cross-reactivity against diverse viral strains. With this in mind, reconstruction of a genotype 1a ancestral genome was achieved using 390 full-length 1a sequences and the encoded glycoproteins of this minimally diverse synthetic isolate (Bole 1a) were successfully incorporated into HCVpps, mediated efficient entry into hepatoma cells and could be blocked by α-CD81 antibodies [89]. Furthermore, CD8+ T-cells expanded with this 1a ancestral sequence were able to recognize epitopes from highly divergent HCV strains, with better coverage than CD8+ T-cells expanded with either a consensus sequence or with individual strains [90]. In conclusion, continued sampling and cataloging of viral sequences will be required to facilitate ongoing characterization of global *E1E2* diversity and these data can help to inform rational immunogen design.

### 3.2. Intrahost Diversity

HCV diversity extends to the intrahost level, with the virus existing within an infected individual as a cloud of related yet genetically distinct variants. In addition to traditional clonal sequencing, recent innovations in sequencing technologies have facilitated more in-depth analyses of viral intrahost populations. The development of single genome amplification (SGA) has allowed recovery of individual sequences from the viral population, enabling assignment of linkage between polymorphisms on individual genomes [91]. However, this method is highly labor and reagent intensive, limiting the depth of population sampling. Next-generation sequencing (NGS) allows viral populations to be scanned at unprecedented depth, two to three orders of magnitude greater than either clonal or SGA strategies [72]. Together these techniques have advanced our understanding of HCV intrahost evolution.

#### 3.2.1. Transmission

Detailed analyses of multiple acute cohorts have shed light on the dynamics of virus envelope glycoprotein diversification during this defining stage of infection. Although viral *E1E2* heterogeneity is detectable at every stage of clinical infection, sampling of recently infected individuals reveals that a genetic bottleneck occurs upon viral transmission, resulting in highly restricted envelope glycoprotein diversity in a newly infected host. Infection can be initiated by a single viral particle or by multiple variants [92], with community acquired infections (transmitted via intravenous drug use) often associated with transmission of multiple variants [93]. Sampling a cohort of 17 acute infections, the number of transmitted/founder (T/F) viruses which established a productive infection was shown to range between and 1–37 viral variants (median 4), with viral transmission from acutely infected individuals resulting in a higher numbers of founder viruses detected in the recipient [94]. Analysis of viral sequences from 18 acute-phase HCV infections revealed productive infection was initiated by >1 T/F strain in 14 individuals, with apparently random sequence diversification in early infection in the HCV ORF [95]. Differential patterns of innate immune induction by T/F strains were also observed, and differed between liver cell-type and infecting HCV genotype [96]. Comparison of *E1E2* sequences from chronic infection with T/F *E1E2*s failed to identify molecular signatures associated with transmission [95]. However, to unequivocally identify T/F strains and identify envelope glycoprotein determinants associated with effective transmission requires analysis of *E1E2* sequences derived from *both* donor and recipient.

Experimental transmission of a defined HCV inoculum to uPA/SCID mice identified 4 key residues in both E1 and E2 selected for upon transmission to a new host [100]. While detectable in the donor inoculum at low frequency, T/F variants lacking a potential N-linked glycosylation site (PNGS) in E2 (N448D) were associated with more efficient target cell entry and selectively out-grew competing variants post-transmission, becoming the dominant circulating species [100]. These T/F variants were not more sensitive to neutralization by an anti-CD81 binding region nAb than dominant donor strains. Analysis of healthcare worker needle-stick-injury transmission events demonstrated between 1–6 *E1E2* variants established initial natural infection in humans and identified signature amino acid substitutions in E2, which differed between donor and recipient, including evidence for parallel diversifying selection at *E2* HVR1 residue 394 in all transmission events [101]. These data also showed that donor sera antibody escape mutants did not predominate post-transmission, although transmitted variants were sensitive to neutralization by human anti-E2 mAbs [101].

HCV can also be transmitted from mother-to-child and vertically transmitted strains exhibit increased viral fitness as a consequence of reversion of CTL escape mutations [102]. Tracking of consecutive vertical transmissions revealed evidence for CTL escape at 2 residues in the E2 glycoprotein [102]. Of note, immunomodulatory changes during pregnancy reduced CTL pressure and facilitated emergence of E2 R495T escape mutants which were not recognised by MHC class I peptide B*5101 492/9, although this substitution significantly impaired virus production. Subsequent conservative R495K mutations facilitated CTL escape while having no deleterious effect in virus particle production [102]. Additionally, a transmission bottleneck is also expected in a liver transplantation setting, where universal re-infection of liver grafts with circulating virus occurs post-transplantation. However, up to 30 independent *E1E2* lineages in a single patient were detected re-infecting a newly transplanted liver [103]. Graft re-infection is characterized by preferential outgrowth of *E1E2* variants that efficiently enter target cells and are resistant to neutralization by pre-transplant sera. This phenotype is mediated by E2 region 425–483 [104]. Together these data indicate that a genetic bottleneck is observed during HCV transmission, with a low multiplicity of related strains transmitted in the majority of cases [92,93,94,96,101]. However, HCV transmission also appears to select for *E1E2* sequences possessing increased replicative fitness, mediating efficient target cell entry and/or escaping host immune recognition [100,101,102,104]. Paradoxically, in some settings, neutralization resistant viruses appear to be preferentially transmitted.

#### 3.2.2. Acute Infection

Sequence diversification from founder strains is gradual in early infection, with a relatively slow nucleotide substitution rate of 2.5 × 10^−5^ mutations per site per genome replication [105]. The acute phase of infection is characterized by limited envelope glycoprotein diversity as a result of the restricted number of T/F viruses, which establish initial infection [92,93,94,96,100,101]. Contrastingly, HVR1 sequence analysis of a limited cohort of newly infected individuals revealed co-infection with multiple viral subtypes and genotypes in acute HCV infection [106]. However, this phenomenon is not widely described indicating it is not a common occurrence and requires determination of sample provenance to rule out cross-contamination [107,108].

Differential patterns of substitutional evolution in *E2* coding-regions have been shown to be predictive of outcome of infection, with resolving acute infection associated with evolutionary stasis, while progression to chronicity was associated with substitutional evolution, primarily focused in HVR1 and coincident with antibody seroconversion [109]. Indeed, higher *d*_N_/*d*_S_ ratios in HVR1 sequences derived from patients with persistent infection are observed when compared to HVR1 sequences from spontaneous resolvers [110]. Re-analysis of both sequence data sets using likelihood-based methods to identify individual amino acids under diversifying selection revealed a significant association between numbers of positively selected sites (*d*_N_/*d*_S_ > 1) in *E2* HVR1 and disease outcome. The highest number were identified in rapid progressors and the fewest in fulminant hepatitis cases, although no specific *E1E2* mutations determined persistence [111]. Together these studies indicate detection of the evolutionary footprints of selective pressure from the host in *E2* HVR1 can predict clinical outcome of infection, and identified HVR1 as a potential immunological decoy [109,110].

The contribution of CTL pressure to *E1E2* diversification in initial infection is multi-factorial, dependent on infecting viral strain and immunogenetics of current and previous hosts. Bull *et al.* report sequential bottlenecks shape acute infection, the first associated with limited number of transmitted viruses and the second at approximately 100 days is associated with a decline in viral diversity and reduction in viral load and correlated with selection at known CTL (G483D and T542I) and B-cell (Y443H/I) epitopes in E2, in addition to others throughout the viral ORF [112]. Experimental infection of chimpanzees revealed that the majority of non-synonymous substitutions observed in acute phase infection are located in class I epitopes, indicating that early viral evolution in the primate model is driven by CD8+ T-cell selection [113]. However, the impact of CD8+ T-cell responses on HCV whole genome evolution in acute infection of humans differs significantly. Kuntzen *et al.* report 30% of all mutations outside E1E2 represent CD8+ T-cell escape mutations/reversions. Contrastingly, no CD8+ T-cell escape was detected in E1E2, although T-cell reversions constituted 40% of all mutations located in the glycoprotein coding-regions, indicating differences in the evolutionary pressures which shape different regions of the viral genome in acute infection of humans [114]. T/F viruses were recently reported to rapidly escape from CD8+ T-cell targeting in initial infection, although most of this targeting occurred outside E1E2 [115]. In two patients who progressed to chronicity, multiple mutations in E1, E2-HVR1 and E2 evolved away from T/F sequences and became fixed in the viral population. However, only one of these mutations was located in a class I HLA epitope, suggesting T-cell pressure is only a minor contributor to *E1E2* sequence evolution in early infection [115]. CD8+ T-cell responses declined rapidly in the two patients who progressed to chronic infection, while viral clearance was associated with maintenance of CD8+ T-cell responses in a single patient.

In tandem with selection or reversion at CTL epitopes, host antibody targeting of the E1E2 complex contributes to glycoprotein sequence evolution in early infection. The post-seroconversion polyclonal antibody response generated to E1E2 in HCV infection targets a broad spectrum of epitopes (detailed in Section 4). Dowd *et al.* report the occurrence of amino acid substitutions throughout E1 and E2, the majority localized in HVR1, and demonstrate evolution and clearance of E1E2 variants driven by host nAb pressure and a correlation between nAb titres and viral clearance. Mutagenesis of specific E2 HVR1 residues dramatically reduced neutralization sensitivity, indicating the majority of the neutralizing response in acute infection is targeted at E2 HVR1 [116]. Indeed, early and broad nAb responses are associated with viral clearance, while patients who progress to chronicity had significantly delayed nAb responses [117,118]. Although no common *E2* sequence determinants associated with resolution of infection were identified, HLA-DQ polymorphisms were associated with breadth of the nAb response [117].

#### 3.2.3. Chronic Infection

Acute HCV spontaneously resolves in only 20% of infections, with the remainder of infections progressing to a chronic viremia. In chronic HCV infection, random seeding of the liver from the periphery occurs which then spreads locally, characterized by transient clusters of 4–50 infected hepatocytes [119]. Patterns and mode of HVR1 evolution in acute HCV can be predictive of progression to chronicity or infection resolution [109,110,111]. Chronic HCV infection is also associated with differential rates of disease progression, with some patients progressing rapidly to cirrhosis and death while others follow a stable disease course with limited liver damage. Rapid disease progression was characterized with persistent high serum ALT levels in conjunction with the profibrogenic chemokine MCP-1, elevated intrahost *E1E2* and HVR1 diversity and a higher rate of synonymous evolution throughout *E1E2*, when compared to mild non-progressive disease [120]. In contrast slow progressors exhibited significant induction of IFN-γ and MIP-1β and increased numbers of positively selected amino acids in HVR1. Patterns of substitutional *E1E2* evolution in perinatal infection have also been shown to be predictive of levels of hepatic injury. The emergence of a diverse HVR1 intrahost population was coincident with antibody seroconversion and associated with limited hepatic injury. In contrast, a mono- or oligoclonal HVR1 population was correlated with high serum ALT levels indicative of increased liver damage [121]. This trend was not correlated with infecting genotype or viral load. In genotype 1 chronic infections substitutional evolution of *E1*/*E2* HVR1 regions to differed markedly according to disease status, with slower HVR1 evolution and divergent evolution of *E1* observed in patients with severe liver disease, when compared to virus derived from patients with mild disease [122].

Longitudinal sampling of *E1E2s* derived from HCV genotype 1a and 3a infections revealed patient-specific signatures of adaptive evolution over the course of chronic infection [123]. However, commonalities between patient specific evolutionary trends were also observed with the majority of *E2* sites subject to strong purifying selection (*d*_N_/*d*_S_ < 1) implying strong structural/functional constraint. Diversifying selection (*d*_N_/*d*_S_ > 1) was detected at a subset of sites in *E2* HVR1, a region targeted by CD81 inhibitory mAbs, a region proximal to the first discontinuous CD81 binding region and putative CD8+ T-cell epitopes. In contrast, no significant evidence for diversifying selection in E1 was detected [123], indicating a lack of host immune targeting of this viral protein in chronic infection. Tracking HCV dynamics in chronic infection from a single genotype 4a-infected patient revealed a complex virome, with subpopulations of variants in direct competition and isolation of IgG-bound virions coincident with the elimination of specific viral subpopulations [124]. Furthermore, detection of low-level recombination events between co-circulating lineages were detected at the *E1*/*E2* junction, implying an additional evolutionary mechanism by which HCV can inflate intrahost *E1E2* complexity [124].

Phylogenetic analysis of *E2* HVR1 variants from four chronically infected patients revealed temporal variation in clustering of sequences, which was correlated with sampling time. Intrahost variability was characterized by a single heterogeneous population in the first three years of infection followed by diversification into multiple subpopulations. While differences in the evolutionary trajectories of HCV populations were observed between patients, all patients *E2* HVR1 sequences exhibited an increase in negative selection over the course of infection [125]. Longitudinal sequence evolution was followed in multiple HCV chronic infections derived from a single source exposure, with patterns of sequence evolution differing between hosts [126]. Phylogenetic analysis revealed mixing of sequences derived from multiple late-infection timepoints (20–25 years post-infection) in some patients, although the majority exhibited clear separation between samplings indicating continued substitutional evolution and lineage replacement. *E1E2* evolution in response to CTL pressure was absent in late infection with purifying selection the dominant force shaping *E1E2* diversity. In contrast, acceleration of HVR1 evolution in late chronic infection was apparent, indicating continued antibody targeting of this immunodominant epitope [126].

Host cellular and humoral responses are considered to be one of the main driving forces shaping HCV envelope glycoprotein diversity in chronic infection. Increasing titre and expanded targeted nAb reactivity to E1E2 is reported over the course of chronic HCV infection [127]. Viral *E1E2* evolution in response to host nAb and CTL targeting was monitored in a single genotype 1a infected patient, revealing dynamic co-evolution at the host-pathogen interface [128]. While strain-specific nAbs against HVR1 were prevalent in early infection, these were replaced by cross-reactive nAbs in later infection. However, despite constant refocusing of humoral responses against current circulating strains, the virus constantly outpaces host adaptive immunity, with mutations in E1E2 neutralizing epitopes rendering nAbs ineffective [128]. Indeed, the majority of sequence changes identified in *E1E2* over chronic infection were located in defined identified T-cell or nAb epitopes. This contrasts starkly with the genomic locus encoding the NS3 protease domain, where no evidence for non-synonymous evolution indicative of viral escape from host responses is observed over the course of chronic infection [129]. After intrahepatic inoculation of two chimpanzees with clonal HCV (genotype 1a), HCV genome evolution was monitored for over four years. Selective pressure in *E1E2* was greatest in acute infection, decreased as infection progressed and was correlated with an increase in anti-E1E2 antibody titers [130]. In contrast to studies of human infection, *E2* HVR1 was relatively stable throughout the course of infection despite the appearance of anti-HVR1 antibodies. Together, these studies indicate substitutional evolution of *E1E2* coding-regions in chronic infection varies between patients and that diversification is partly driven by adaptive immune responses. Additionally, differences in viral diversification between humans and chimpanzees are apparent.

## 4. Immunogenic Regions of E1 and E2

The E1 and E2 proteins are immunogenic in natural infections and following experimental immunization of animals. Their presence on the viral surface, as well as roles in cell binding and fusion make them the main targets of neutralizing antibodies. It appears that E1 is less immunogenic than E2 in natural infection, with relatively few reports of monoclonal antibodies to the E1 protein [131,132]. However, immunization with E1 results in the generation of antibodies [133] and some chronic infections result in antibodies to a conserved epitope between aa313–327 [134]. By contrast, there is extensive evidence for antibodies to the E2 protein in HCV infections. Early studies performed in a chimpanzee infection model supported the hypothesis that the neutralizing antibody response elicited during acute and chronic infection targeted mainly the HVR1 region [135], resulting in positive selection of neutralization-resistant members of an infecting HCV quasispecies [109]. HVRs are under constant immune selection, acting as decoys that shield the more conserved receptor binding regions from antibody recognition [55,136]. Antibodies targeting these regions are often, but not always, strain-specific [135] and conformation sensitive [137,138]. Antibodies targeting the N-terminus of HVR1 are non-neutralizing [12], while those recognizing the C-terminal region of HVR1 demonstrate restricted neutralization [12,137,138]. This region is therefore likely to be an undesirable target for vaccines due to their restricted breadth of the antibodies produced.

**Figure 5:**
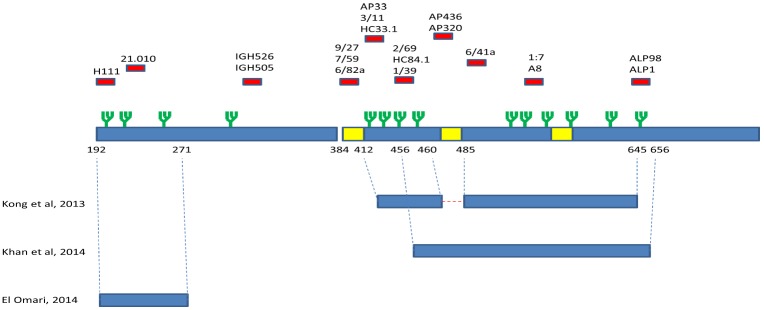
Cartoon of the protein constructs used for crystallization of E1 (71) and E2 [42,49] mapped onto the HCV E1 and E2 genes (blue). Regions possessing antibody epitopes are highlighted in red; glycosylation sites are indicated by a green branching structure; and hypervariable regions are highlighted in yellow. The numbering of the ends of the protein constructs are highlighted. A red dashed line represents a Gly-Ser-Ser-Gly linker added between two regions in the E2 core construct reported by Kong *et al.*, 2013 [42].

**Figure 6:**
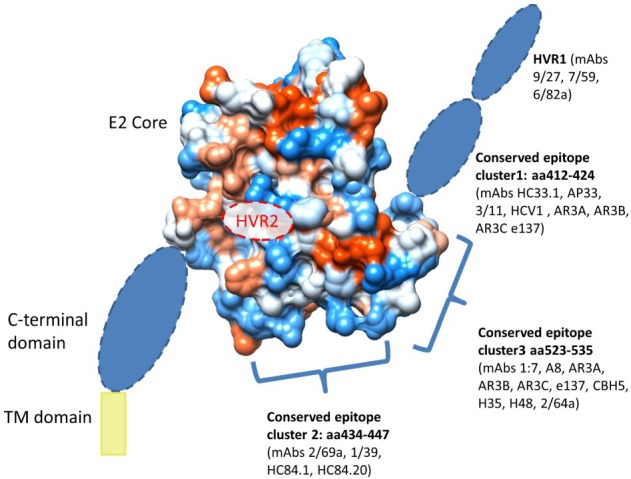
Proposed structure of the E2 protein, highlighting hyrophobic side chains (red) and hydrophilic side chains (blue) on the conserved E2 core crystal. Unresolved regions including the conserved N-terminal domain, HVR1 and the unresolved region of the ectodomain (aa646–715) are highlighted as blue ovals. The G-S-S-G linker used to replace the HVR2 in the structure presented by Kong et al is identified by a dashed red oval labelled “HVR2”. The transmembrane domain (aa 716–745) is highlighted in yellow. Three separate linear antigenic clusters are highlighted, which make a contiguous antigenic region that possesses many antibody epitopes (reviewed in Edwards *et al.*, 2012 [152]). Most of these epitopes are linear and not dependent of the overall conformation of the E2 core. Some antibodies (such as AR3A, AR3B, AR3C, and e137) recognize overlapping conformation-dependent discontinuous epitopes.

In addition to the HVRs, it has now been established that many regions of E2 are immunogenic and possess epitopes recognized by neutralizing antibodies, particularly the discontinuous region surrounding the CD81 binding site. Alternative approaches have yielded very similar results: Antibodies isolated from human B cells were used to identify neutralizing antibodies overlapping the CD81 binding site [139,140,141,142,143,144]. Antibodies isolated from phage-displayed libraries have also yielded antibodies with similar, overlapping, specificities [145,146,147,148,149]. The conserved nature of these epitopes is an indication that this antigenic region is under functional constraint and that entry absolutely requires interaction with CD81. More recently, conserved neutralization epitopes have been described that are discrete from the CD81 binding region [138,150]. From this we have built an antigenic model of the E2 protein that is consistent with the crystal structure of the E2 core [151]. Together, the clustering of known antibody epitopes on E2 confirms that a broadly conserved neutralizing face exists around the discontinuous CD81 binding site, with the amino acids 412–424, 436–442, and 520–535 possessing contact residues for many neutralizing antibodies. mAbs AP33, 3/11, HC33.4, HCV1 and H77.39 all recognize contact residues in the region 413–420. mAbs 2/69, HC84.1, HC84.27, 1/39, mab#8 and mab#12 have epitopes in the region 427–447, while mAbs H77.31, H77.36, 1:7, A8 have epitopes in the region 523–535. Some neutralizing antibodies have epitopes spanning multiple conformation-sensitive regions, in particular mAbs AR3A, AR3B, AR3C and e137 (reviewed in [152]). In addition, there is an exposed face that elicits neutralizing antibodies with restricted activity, and a discrete “non-neutralizing” face defined by the epitope recognized by the mouse monoclonal antibody H53 [24,152] (see Figure 5 and Figure 6).

The generation of antibodies to E1 and E2 were thought to be delayed and to not contribute to spontaneous resolution of viremia in acute infections, both in chimpanzee models of infection and natural human infections [127,153]. However, accumulating evidence supports the hypothesis that a particularly broadly active neutralizing antibody response soon after infection might contribute to prevention of chronic infection [116,118,154,155]. It is clear that the majority of people infected with HCV do not generate broadly neutralizing antibodies until chronic infection is established, where anti-E1/E2 antibodies are readily detectable [156,157]. Interestingly, the component of the antibody response in chronic infection directed to conformation-sensitive epitopes is more broadly reactive than those antibodies recognizing contiguous epitopes [156], suggesting that the conserved epitopes accessible on the surface of virus particles are generally conformation sensitive. This contrasts with antibodies generated by experimental immunization with recombinant glycoproteins constructs, which are commonly directed to linear epitopes and do not cross-neutralize diverse HCV strains [12,158,159]. Thus a vaccine might have to present complex conformational epitopes to successfully produce cross-reactive antibodies. The breadth of neutralization of the polyclonal response in chronic infections is not defined by the genotype of the infecting isolate [156], suggesting that the individual host antibody repertoire defines the specificity of the antibodies produced.

While we still do not understand the exact epitope specificity of antibodies associated with spontaneous resolution of infection, it is plausible that the conserved N-terminus of E2 is a key target. Screening the sera of patients with either acute and chronic infection for reactivity to peptides representing this region suggested that the prevalence of antibodies to this region is low, and similar in both acute and chronic infection [60], but with the subsequent discovery of multiple conformational states of this region it is possible that antibody reactivity to a preferred conformation might be associated with improved ability to neutralize virus entry. Naturally-occurring escape mutants in this region are rare [160], and selection of escape mutants *in vitro* resulted in significant loss in entry fitness [161]. However, presentation of this epitope is dependent on the global conformation of E2, and antibodies recognizing an epitope in the region aa 434–447 have also been found to specifically block binding of these neutralizing antibodies and interfere with their neutralizing potency (see Section 4.1). A vaccine based on this partially conformation-sensitive region fixed in an optimal β-hairpin fold might preferentially induce potently neutralizing antibodies, but would also have to avoid inducing interfering antibodies.

Immunization with glycoprotein constructs has resulted in varying degrees of success in generating neutralizing antibodies. Some immunization protocols have generated broadly neutralizing antibodies in humans and experimental animal models [162,163,164], while others failed to generate a broadly neutralizing antibody response [165,166]. However, differences in immunization protocols, adjuvant selection and virus strains used for neutralization assays could account for these differences. These discrepancies highlight the need to standardize protocols for and assessment of neutralizing antibody responses. However, it is clear that choice of adjuvant is important to determining the quality of antibody response in vaccinees [162].

Early studies generating murine monoclonal antibodies generated both neutralizing and non-neutralizing murine monoclonal antibodies [12,159]. Neutralizing antibodies have been raised with the E1 protein, in the absence of a neutralizing antibody response when using an HVR1-deleted E2 protein [167]. We do not know how the characteristics of these responses compares between studies, but experiments with phage displayed libraries of nanobodies revealed that the most potent neutralizing nanobodies target epitopes overlapping the CD81 binding site [163]. Additionally, immunization of goats and humans with an E1/E2-based vaccine resulted in antibodies that compete with CD81 binding site mAbs AP33, 1:7, HC33.4, HC84.26 and AR3B, and neutralized entry of some isolates. Improved understanding of the molecular structures of key epitopes make these promising targets for vaccine design [61,62,63,65,146,168,169].

### 4.1. Mechanisms for Evasion from Neutralizing Antibodies

As conserved epitopes overlap functionally conserved receptor binding regions, HCV has evolved a number of mechanisms to shield these key epitopes from antibody recognition. A key factor in resistance to neutralization is the highly glycosylated nature of the E1 and E2 proteins. E1 possesses between 4 and 5 N-linked glycosylation sequons (NXT/S), while E2 possesses between 9 and 11 sites [30,170]. In addition to shielding key epitopes from antibodies [66,171,172], these glycans may by essential for interaction with CD81 [29]. As such they are the target for lectins that interact with mannose [30,173,174] or N-acetylglucosamine [175,176] present in the conserved oligosaccharides on the surface of the proteins. Three of the glycans associated with E2 modulate neutralization by antibodies binding the CD81 binding region [170], highlighting their role in protecting the CD81 binding site.

HCV glycoproteins also interact directly with apolipoproteins [56,74], which are essential for assembly and release of virus particles from infected cells [177], as well as assisting cell attachment [178] and membrane fusion [179]. It is possible that these lipoproteins protect neutralizing epitopes on the surface of E1 and E2 [58,75,157,180,181], covering these epitopes until initial binding events with a cell surface lead to revealing of the CD81/SCARB1 binding sites [56]. They also inhibit recognition and neutralization by ficolins [176], and present epitopes for virus-neutralizing antibodies directly on the apoliproproteins [56]. Together this demonstrates a dynamic interplay between lipoproteins, protein epitopes and glycans with adaptive and innate components of humoral immunity.

An additional mechanism to avoiding antibody-mediated neutralization is the ability of HCV to directly transmit between hepatocytes, avoiding exposure to extracellular antibodies [182,183,184,185,186]. However, it appears that some epitopes are accessible to virus being transmitted by this process, particularly those that overlap interactions with CD81. Antibodies targeting the virus [163,164] or receptors [187,188] can inhibit cell-cell transmission, suggesting that immunotherapy might prevent spread of infection, especially of strains resistant to other antiviral therapies [189].

Antiviral antibodies do not always contribute to clearance of infection. The discovery of antibodies that can interfere with the action of neutralizing antibodies [190,191], or even enhance infection [156,192,193] highlight the importance of targeting vaccines to specific, broadly conserved neutralization epitopes. Antibodies targeting the regions aa 434–447 in the ectodomain of E2 have been described to either neutralize HCV entry [12,61,194] or interfere with the action of other neutralizing antibodies, particularly those targeting the conserved region between aa 412–424 that possesses epitopes for many neutralizing antibodies [191,195]. This discrepancy has been explained by recent analysis of the structure of this region in complex with different monoclonal antibodies. Neutralizing and interfering antibodies were found to occupy alternative orientations when in complex with a peptide representing this region [196]. These interactions occur with conformationally discrete forms of E2, which may represent alternative steps in the virus’ life cycle, or imply induced fit occurring following interactions between peptide epitopes and the respective antibodies. Nonetheless, these studies highlight the need to understand the antigenicity of the entire protein, rather than just individual epitopes, as there may be conformational elements to linear regions of these proteins that define the quality of the antibodies produced following immunization.

The discovery of infection-enhancing antibodies poses an additional problem for vaccine design. In a study of the neutralizing properties of polyclonal antibodies purified from chronic infections, IgG preparations were found to neutralize some HCV isolates, yet enhance infection of others [61]. This suggests that a difference in epitope exposure defines a virus’ sensitivity to antibody-mediated neutralization or enhancement. The mechanism underlying this apparent enhancement is unclear. Expression of the hepatocyte-expressed FcRn Ig receptor appears not to have a role in HCV-mediated mediated neutralization [197], but might have a role in increased uptake of HCV with specific antibodies. Alternatively, conformational changes brought about by antibody interactions might contribute to enhanced receptor interactions and fusion. The close relationship between receptor binding, neutralization sensitivity and infectivity has been demonstrated [55,75], and it is plausible that antibody-induced conformational changes in E2 contribute to enhanced infectivity. Together, these antibody evasion mechanisms present a significant challenge to vaccine design. A targeted, immunofocussed immunogen presenting genetically and structurally conserved regions of the E1 or E2 proteins to the immune system may be able to elicit antibodies capable of neutralizing genetically diverse strains of HCV, but such an immunogen must avoid the production of interfering, non-neutralizing, or infection-enhancing antibodies.

## 5. Glycoprotein Functionality

HCV glycoproteins facilitate entry of virions into target cells and play an important role during production of infectious viral progeny. To fulfill these tasks, gross conformational changes within these proteins must occur in a controlled and coordinated manner, requiring their close interaction and co-evolution. Additionally, HCV glycoproteins utilize a defined set of host factors for these processes. Thus, even though the immune system puts strong selection pressure on the viral glycoproteins to change their sequence over time, conserved functions and host factor interactions put constraints on their variability.

### 5.1. Entry

HCV entry requires at least four crucial host factors: SCARB1, CD81 and the tight junction proteins Claudin (CLDN) and Occludin (OCLN) [13,14,198,199]. In addition, multiple accessory factors have also been implicated in the entry HCV process [200,201,202,203,204,205,206,207,208]. In the serum, HCV circulates as lipoviral particles, similar to very-low-density-lipoprotein (VLDL) particles. Thus; not only the viral glycoproteins themselves but also the lipoprotein moiety of the HCV particle influences viral entry [178,209,210,211,212]. Indeed, SCARB1 and LDL are lipoprotein receptors, which interact with apolipoproteins including ApoE, which is known to associate with HCV particles [213,214,215,216].

Initial studies aimed to elucidate the conservation of host factor usage for particle entry among HCV genotypes. Before chimeric HCVcc particles harboring structural proteins from all HCV genotypes were available, HCVpp were employed as a model system. Lavillette and co-workers demonstrated that HCVpps bearing glycoproteins from genotypes 1–6 all entered cells by endocytosis and depended on acidification of endosomal vesicles for fusion [11]. All isolates utilized CD81 with comparable efficiency, while efficiency of usage of SCARB1 seemed to be more variable. These data were subsequently confirmed with chimeric HCVcc particles, in which the regions encoding core to NS2 were derived from genotype 1–7, while the remaining viral proteins were derived from the genotype 2a JFH1 isolate which efficiently replicates in cell culture [217]. All genotypes showed comparable sensitivity to neutralization with CD81-specific antibodies, while inhibition of entry by SCARB1 specific antibodies again revealed variability between isolates.

While the envelope glycoproteins interact directly with CD81 and SCARB1, the interactions with CLDN1 and OCLN are less well defined. CLDN-1 was initially identified as an important host factor for HCV entry [198] and subsequent studies revealed that CLDN-6 and 9 can also facilitate HCV entry [218,219]. However, isolate-specific differences in the breadth of CLDN-usage were uncovered utilizing chimeric HCVcc viruses [220]. While all genotypes are able to utilize CLDN1 for cell entry, only some isolates also had the ability to switch to CLDN 6 or 9 if CLDN1 was absent or if CLDN1 accessibility was impaired due to addition of CLDN1-specific antibodies [220]. Furthermore, CLDN1-dependent viruses can be adapted to utilize CLDN6 or CLDN9, with an individual amino acid exchange in E1 sufficient to confer this phenotype [221]. Interestingly, while CLDN1 mRNA could be detected to similar levels in liver biopsies of HCV infected patients, CLDN6-specific mRNA abundance was highly variable between patients [220]. Additionally, utilizing mutant OCLN proteins harboring epitope insertions within their extracellular region, Sourisseau and co-workers reported differential usage of OCLN by individual HCV isolates, supporting a model where HCV directly interacts with OCLN [222].

Viral adaptation experiments have further elucidated the functional impacts of HCV glycoprotein variability. After long-term passage in cell-culture, a viral variant of the genotype 2a isolate JFH1 was identified that harbored a G451R amino acid exchange in E2. This resulted in aberrant density distribution of particles after iodixanol gradient centrifugation, indicative of changed association with host cell-derived lipids and lipoproteins [75,223]. Furthermore, viruses carrying this mutation were found to be less susceptible to neutralization by CD81 antibodies and to have a reduced dependency on SCARB1. A similar phenotype was observed due to an I414T mutation in E2, which was identified in a different experiment [224]. However, as particle density distribution was affected by both mutations, the effects on SCARB1 usage during virion entry could also be indirect due to differences in lipoprotein and apolipoprotein loading on virions harboring mutant glycoproteins.

While deletion of HVR2 and the IgVR are lethal for the virus, virions with a deletion of HVR1 in E2 (ΔHVR1) retained infectivity *in vivo* and *in vitro*, but with impaired specific infectivity [55,225,226]. Genotype 2a chimeric viruses (Jc1) possessing ∆HVR1 modifications reduced the abundance of HCV particles with very low buoyant density and ablated their infectivity, hinting at changes in lipoprotein association [55]. Furthermore, ΔHVR1 viruses were less dependent on SCARB1 for entry, while CD81 usage was unaffected. Additionally, ΔHVR1 viruses were more susceptible to neutralization by E2-specific antibodies, confirming that this HVR shields important conserved epitopes in wild-type E2. In a follow-up study, it could be shown that the aberrant density profile of Jc1-derived ΔHVR1 virus was not due to differences in the amount of ApoE on these particles. However, it seems that the conformation of this host factor was changed, which may also affect the interaction with SCARB1 [56]. Other studies determined that deletion of HVR1 could also influence interaction with the LDLR and led to a more pronounced impact on viral infectivity in some genotypes [227,228].

While the high variability of HCV severely hampers the development of a protective vaccine, the ability of glycoproteins to adapt to different environments can also be exploited in HCV research. Although recent technological advances have resulted in the development of multiple *in vivo* models for the study of HCV [229], a fully immunocompetent small animal model remains elusive. Previous studies demonstrated HCV tropism is partially defined at the level of viral entry due to species-specific usage of CD81 and OCLN [199,230,231]. The mouse variants of these receptors can only be employed very inefficiently. To overcome these blocks, the HCV chimera Jc1 was adapted to utilize mouse CD81 (mCD81) [232]. This phenotype was achieved with one mutation in E1 and two mutations in E2. Interestingly, additional analyses determined the adapted glycoproteins could use mouse OCLN and that HCVpp harboring glycoproteins from the adapted virus were able to enter murine cells in the absence of human entry factors. Furthermore, these mutations also enabled utilization of rat and hamster CD81 and decreased susceptibility to neutralization with CD81-specific antibodies. However, neutralization with E2-specific antibodies was increased, demonstrating a fitness cost associated with adaptation. Recently, a follow-up study determined that this variant was also able to infect pigtail macaque-derived hepatic cells from induced pluripotent stem cells, further advancing the development of small animal models for HCV [233]. In summary, HCV glycoprotein variability affects functionality during the entry cascade and also influences the interaction of virions with lipids and lipoproteins.

### 5.2. Assembly and Release

Although the NS HCV proteins are integral for virion assembly [234,235,236,237,238,239,240,241], the structural proteins core, E1 and E2 also play crucial roles in the production of infectious viral progeny. Both viral and host factors are essential for virion assembly and release. The virus hijacks the host-cell lipid machinery for particle production, utilising factors that are involved in the secretion of lipoproteins, including the microsomal triglyceride transfer protein (MTTP), in addition to apolipoproteins B and E (ApoB, ApoE) as well as other exchangeable lipoproteins [216,242,243]. ApoE plays a major role in HCV assembly via interaction with the viral glycoproteins [33,186,216,244,245]. Related apolipoproteins can be utilized for virion assembly in the absence of ApoE, at least for genotype 2a, 1b and 3a, indicating that utilization of exchangeable lipoproteins is relatively conserved among HCV genotypes [246]. However, as glycoprotein diversity influences host factor usage during viral entry, it is likely that this diversity will also translate into differences in ability to interact with host factors during particle production.

The generation of chimeric and recombinant viral genomes, replacing glycoprotein regions with corresponding regions from different isolates and genotypes, has uncovered important functions of the HCV envelope proteins during virion morphogenesis. Characterization of domains from E1 and E2 indicated that these proteins have co-evolved together within specific genotypes to facilitate optimal interactions between the two proteins, in addition to conferring efficient virion assembly, release and entry [247]. Characterization of HCV recombinants with chimeric E1/E2 complexes was performed using genotype 1a/2a JFH1-based recombinants expressing 1a core-NS2, where E2 sequences were exchanged with sequences from functional isolates of genotypes 1a, 1b, and 2a [248]. This study identified specific amino acid residues in the stem region of E2, which are important for both HCV entry and for production of infectious virions. Combining E1 from genotype 2a and E2 from genotype 1a in the HCVpp system resulted in non-functional particles and it was observed that the stem region of E2 is an important segment for entry. However, HVR2 and the IgVR as well as another segment in domain II of the HCV glycoproteins were shown to be crucial for assembly in the HCVcc system [17]. Intra-genotypic chimeric HCV genomes revealed that core and p7 are also critical determinants for viral particle production, and the glycoprotein chimeras between two genotype 2a isolates (J6CF and JFH-1) were fully functional and generated particles with comparable efficiencies [249]. Contrastingly, HCV assembly of inter-genotypic glycoprotein constructs was not efficient, and the swap of genotype 1 glycoproteins into a genotype 2a genome resulted in the accumulation of non-enveloped core protein structures, demonstrating an important role for the glycoproteins in viral envelopment [249]. These results suggest that genetic differences between viral isolates from divergent viral genotypes preclude efficient virus production from inter-genotypic chimeric genomes, but the exchange of viral glycoproteins within a given viral genotype may be tolerated. Recently these findings were confirmed in a *trans*-complementation approach for all HCV genotypes, demonstrating glycoprotein-dependent production of infectious particles in a genotype- and isolate-specific fashion [19]. This new cell culture system also allowed the incorporation of primary envelope protein gene from genotype 1 and 2, allowing dissection of glycoprotein gene function in an individualized fashion [19].

### 5.3. Interferon

In addition to their functions in humoral immune evasion, virion entry and infectious particle production, HCV envelope proteins have also been implicated in interfering with host innate immunity. Single nucleotide polymorphisms (SNPs) associated with the *IFNL3* (*IL28B*) locus or indels, which produce the *IFNL4* gene, profoundly influence response to interferon (IFN)-based therapies and the outcome of HCV infection [250,251]. One mechanism by which IFN-α inhibits HCV is by reducing the infectivity of secreted virions by inducing conformational changes in the E2 glycoprotein, affecting receptor utilization and rendering virions more susceptible to neutralization [252]. Inhibiting the interferon (IFN) signal pathway, which results in a cellular antiviral state via upregulation of hundreds of interferon stimulated genes [253], is therefore clearly advantageous for the virus.

Prior to DAA-based regimens, HCV treatment options were limited in their efficacy and did not target the virus directly. IFN-based therapies promote viral clearance by boosting host immune responses although differential therapy response rates are associated with different viral genotypes. IFN-resistant phenotypes are exhibited by genotype 1 and 4 viruses, which share a common ancestor in Central Africa [79,254]. The IFN-resistant phenotypes exhibited by genotypes 1 and 4 likely evolved historically in response to allelic frequency variation at the *IFNL3* linked SNP rs12979860 in human populations within this region [255]. Phylogenetic reconstruction of the shared ancestor of genotype 1 and 4 viruses identified a total of 82 amino acids that evolved uniquely on the branch ancestral to genotype 1 and 4 virus, which potentially represent candidate mutations contributing to the IFN resistant phenotypes exhibited by these strains. Seventeen of these candidate IFN-resistance mutations are located in E1 and E2 [255].

The E2 coding region possesses a 12 amino acid motif, termed the PKR eukaryotic initiation factor 2 alpha (PKR-eIF-2α) phosphorylation homology domain (PePHD), which is homologous to PKR and its target eIF2a. By binding to PKR, the E2 PePHD inhibits its activity and therefore reduces the antiviral activity of IFN [256,257]. Multiple mutations in the PePHD region are reported to be correlated with responsiveness to IFN-based therapies [258,259] however this association is controversial [260,261,262]. Long term passage of HCV in cell-culture in the presence and absence of IFN results in multiple mutations across the genome associated with both increased fitness and IFN-resistance [263,264,265]. In a genotype 1a (strain H77) and 3a (strain S52) context, E1 mutation I348T and E2 mutations F345V and V414A enhanced viral entry and release and conferred IFN-α resistance [263]. These mutations acted in a genotype-dependent manner but were undetectable in patients who failed IFN therapy. In a genotype 2 context (Jc1) a direct correlation with increased PKR phosphorylation and IFN resistance and enhanced viral fitness was associated with mutations in E1 (T17A) and E2 (S18G) [264]. The HVR1 region itself has been investigated for its role in responsiveness to IFN-based therapies, with some association between HVR1 diversity and early response to treatment reported [266]. The E2 glycoprotein has also been suggested to play a role in inhibiting the production of IFN-α and IFN-γ in plasmacytoid dendritic cells (pDCs) exposed to HCV-infected hepatocytes. E2 binds to (pDC) c-type lectins and induces a rapid phosphorylation of Akt and Erk1/2 in pDCs, thus causing a negative interference of CLR signaling resulting in the inhibition of the principal function of pDCs, the production of IFN [267].

## 6. Vaccine Approaches

Neutralizing antibody responses are critical components of the host defense against viral infections and are recognized as a key element in the protective immune response against infection elicited by many prophylactic vaccines [268,269]. However, most HCV vaccine studies to date failed to elicit cross protective immune responses, therefore recent research efforts have focused on defining conserved immune epitopes and developing novel animal models. In the case of HCV, several lines of evidence support a role of antibodies in the control of HCV infection. Firstly, in acute HCV infection early and robust neutralizing antibody titers are correlated with viral clearance [117,118,155]. Moreover, B-cell depleting rituximab regimens during chronic HCV infection are associated with increased viremia which returns to baseline after reappearance of B-cells [270,271] and spontaneous HCV clearance of chronic infection was accompanied by appearance of neutralizing antibodies [272]. Secondly, several laboratories have isolated a large spectrum of broadly cross-neutralizing human monoclonal antibodies from chronically HCV infected patients [65,131,132,139,142,143,145,146,147,150,194,273,274,275,276,277]. Finally, animal challenge studies involving chimpanzees or human liver chimeric mice have established that administration of antibodies can confer protection from HCV challenge [135,145,278,279] and abrogate established infection [280]. As this review is concerned with envelope glycoprotein structure, in this section we discuss only vaccination approaches geared to generating humoral E1E2 specific immunity, and not the approaches intended for T-cell-based immunity targeting the HCV non-structural proteins.

### 6.1. Recombinant Protein

The first prophylactic HCV subunit vaccine candidate was developed by Houghton and colleagues and based on genotype 1a recombinant E1 and E2 glycoprotein heterodimer (gpE1-E2) purified from cell extracts. In preclinical studies conducted in chimpanzees the vaccine induced high titers of E1 and E2 specific antibodies and elicited a high level of protection against intravenous challenge with the homologous virus. Five out of seven vaccinated animals were sterilized to the challenge as they did not display any markers of infection during follow up. The two additional animals became infected but cleared the infection during the acute phase while all four control animals developed a chronic HCV infection [281]. Protection was correlated with a high titer of anti-E2 abs thus providing substantial encouragement that recombinant HCV E1-E2 proteins may induce robust and protective antibody responses [281]. Subsequent chimpanzee vaccination studies using adjuvanted recombinant E1E2 formulations were conducted, challenging with both homologous and heterologous viruses, and indicating induction of protective but not sterilizing immunity [282,283]. Therefore, from vaccination regimens utilizing homologous and heterologous strains, measurable and clinically relevant protection in the most authentic animal model for HCV was achieved.

Recombinant E1E2 glycoproteins derived from the HCV1 strain (genotype 1a) were immunogenic in rodents and chimpanzees and elicited protective immune response in chimpanzees against challenge with homologous or heterologous genotype 1a viruses [165,284]. Safety and tolerability of this recombinant E1E2 was tested in healthy human volunteers adjuvanted with MF59 (HCV E1E2/MF59C.1), which was well tolerated and able to stimulate cellular and humoral responses [285]. This immunogen was used to immunize 16 healthy human volunteers, however vaccination only induced the production of pan-genotypic nAbs in a single vaccinee [286]. These combined data indicate that recombinant glycoprotein vaccine can elicit cross-neutralizing antibodies targeting broadly conserved epitopes. This conclusion is supported by the production of monoclonal antibodies that have broad cross-neutralizing effect and have shown to be protective against heterologous HCV clades in passively immunized animals [282,283]. A multipeptide therapeutic HCV vaccine without adjuvant, containing three components of the glycoproteins (Cenv3), was tested in a phase II clinical trial on patients who were chronically infected with genotype 4 viruses and were defined as non-responders to IFN-RBV therapy. *In vitro* it was demonstrated that the antibodies generated against the Cenv3 epitopes had a neutralizing effect [287]. This vaccine was safe and did not appear to result in serious adverse events. Cenv3 induced both arms of adaptive immunity with subsequent reduction in viral titers, although it was unsuccessful at clearing HCV [287].

### 6.2. Inactivated Virus

Utilizing well-established procedures for propagation of wild-type HCV in tissue culture, genotype 2 virus was produced, purified and inactivated, followed by injection into BALB/c mice with adjuvant [288]. Antibodies against E1E2 were detected in mouse sera and displayed cross-reactivity against HCV strains, neutralizing infection with genotype 1a, 1b and 2a viruses. Additionally, immune sera from mice also prevented infection of immunodeficient mice with humanized livers and was more efficient at neutralizing homologous virus than immune sera from mice treated with recombinant E2 alone or recombinant E1E2s. These data indicate that E1E2s presented on intact virions are more immunogenic than isolated recombinant glycoproteins [288].

### 6.3. DNA Vaccines

DNA vaccines are easily manufactured and stored on a large scale, and can be produced at greatly reduced cost when compared to protein-based vaccines or inactivated viruses. Both prophylactic as well as therapeutic DNA vaccines have been tested with the HCV glycoproteins as the encoded antigen. Prophylactic DNA vaccination demonstrated that a cell surface expressed E2 protein from a genotype 1a virus could elicit immune responses in mice [289]. Following this, a DNA plasmid encoding a truncated cell surface expressed E2 protein from a genotype 1a strain was injected into chimpanzees intramuscularly. Although this candidate vaccine did not protect chimpanzees from infection after HCV challenge, vaccinated chimpanzees resolved infection while the control animal became chronically infected [290]. A therapeutic DNA vaccine candidate (CIGB-230) was the first to reach a phase II trial and comprises a mixture of a plasmid expressing the HCV structural proteins of genotype 1b isolate, with recombinant HCV core protein delivered intramuscularly. CIGB-230 elicited strong humoral and cellular immunity and protected mice against the challenge with a recombinant vaccinia virus expressing the HCV structural proteins [291]. The Phase I clinical trial conducted with CIGB-230 included patients infected with HCV genotype 1b who failed to respond to previous IFN plus RBV therapy. Although 40% percent of vaccinated individuals exhibited improved or stabilized liver histology with reduced fibrosis, detection of HCV RNA persisted [291,292].

### 6.4. Virus Like Particles

A virus like particle (VLP) is an assembly of virus structural proteins that mimic the configuration of a real virus. This VLP approach in the HCV field has been inspired from the successful application of Hepatitis B virus (HBV) and Human papillomavirus (HPV) vaccines [293,294,295]. Indeed, the baculovirus expression system for the production of VLPs has been approved by FDA and has recently been licensed for HPV vaccine production for use in humans (Cervarix^®^ by GlaxoSmithKline). Insect cells were used for expression and assembly of HCV VLPs, and their expression and immunogenicity determined. The structural proteins of HCV cloned from genotype 2 strains and expressed in insect cells and were able to elicit strong humoral immune response against HCV in mice [296,297].

Production of HCV-like particles (HCV-LPs) containing genotype 1b structural proteins was achieved in insect cells and used for immunization of chimpanzees [298]. Although no significant antibody production was reported, HCV-LPs induced HCV-specific cellular immune responses. Previous studies in mice and baboons demonstrated induction of anti-HCV antibodies, although the antibody titers induced in baboons were low [299]. HCV-LPs induced virus-specific humoral and cellular immune responses in BALB/c mice and HLA-A 2.1 transgenic (AAD) mice [300]. These HCV-LP-induced immune responses protected mice from challenge with recombinant vaccinia virus expressing HCV structural proteins (vvHCV.S) in a surrogate HCV-vaccinia challenge model [300]. Immunization with plasmid DNA forming VLPs pseudotyped with glycoproteins from a genotype 1a strain and/or displaying NS3 antigen in capsid protein induces strong pangenotypic T-cell responses. This vaccine platform is based on retrovirus-like particles made of murine leukemia virus (MLV) gag and pseudotyped with HCV envelope glycoproteins. These retro-VLPs can be produced *in vivo* (plasmo retro VLP) and induced HCV-specific nAbs as well as T-cell and B-cell responses [301].

Chimeric HBV-HCV particles comprising HCV genotype 1a E1 and E2 and HBV S proteins were efficiently co-assembled and presented HCV glycoproteins in an appropriate conformation for the formation of E1E2 heterodimers [302]. Produced in stably transduced CHO cells and used to immunize New Zealand rabbits, these particles induced a strong, specific antibody response against the HCV and HBV envelope proteins in immunized animals. Sera containing anti-E1 or anti-E2 Abs elicited by these particles neutralized infections with HCV pseudoparticles and cell-cultured viruses derived from different heterologous 1a, 1b, 2a, and 3 strains [302]. Using retrovirus-derived VLPs pseudotyped with HCV 1a structural proteins, broadly neutralizing antibodies against HCV in macaques were raised that cross-neutralized HCV genotypes 1b, 2a, 2b, 4 and 5. In this study, priming with adenovirus expressing the glycoproteins, followed by boosting with VLPs pseudotyped with HCV envelope proteins generated broadly neutralizing antibodies [303].

### 6.5. Viral Vectors

The delivery of viral genomes using alphaviruses, adenoviruses or measles virus (MV) all represent potential vaccination strategies. Several preclinical studies targeting ranges of infectious diseases have demonstrated that the combined use of adenovirus prime and protein boost induces high antibody titers [304]. Combining an adenoviral vector prime that was known to induce potent anti-HCV T-cell responses, in conjunction with recombinant genotype 1b E1E2 protein induced nAb responses in mice and guinea pigs, with immune sera from immunized animals broadly neutralizing a pan-genotypic panel of HCVpps and HCVcc *in vitro* [164]. Two reports described cross-neutralizing antibody responses in rodents vaccinated with viral vectors and recombinant envelope protein. Firstly, priming with Th1-adjuvanted E1E2 and non-structural proteins (NS3-5) followed boosting with chimeric, defective alphaviruses expressing these HCV genes elicited the induction of anti-E1E2 antibodies able to cross-neutralizing heterologous HCV strains *in vitro* [305]. In a similar approach, the structural proteins from the HCV genotype 1a were engineered in an MV vector either forming a heterodimer or the glycoproteins separately fused to the cytoplasmic tail of the MV fusion protein. Immunization of MV susceptible mice induced neutralizing antibodies to both MV and HCV. It was observed that, upon boosting with a recombinant soluble E2, induction of cross-neutralizing antibodies was achieved [306].

### 6.6. Passive Administration

Passive administration of anti-E2 nAbs have been shown to protect uPA/SCID mice from heterologous viral challenge [145]. Additionally, humanized mice were also protected from infection with monotypic viral challenge via administration of nAbs, which also abrogated ongoing infection [280]. Chimpanzees passively immunized with rabbit antisera specific for E1 and E2 were protected against HCV challenge [135]. In contrast, chronically infected chimpanzees treated with anti-E2 nAb HCV1 resulted in a reduction in viral load but no resolution of infection [278]. Passive administration of human anti-HCV nAb MBL-HCV1 delayed viral rebound in a post-liver transplantation setting, but did not protect against graft re-infection [307]. Similarly, polyclonal anti-HCV immunoglobulins (Civacir) [308] and anti-E2 mAb (HCV-Ab^XTL^689) [309] administered to liver transplant patients resulted in reduction of HCV RNA levels, but again, no long term protection against liver pathology was demonstrated.

In summary, while many different potential vaccination strategies for HCV exist, none that have graduated to human clinical trials have elicited protective responses. A pan-genotypic HCV vaccine would be a major accomplishment and should incorporate conserved epitopes from the HCV structural proteins in their correct conformations to induce high titers of broadly neutralizing antibodies and should also elicit strong cellular responses. In summary, standardized panels of nAbs and representative E1E2s from diverse isolates and representing a range of neutralization sensitivities should be made available to help define the precise humoral immune correlates of protective antibody responses. Strategies to trigger these protective responses in humans must be developed, facilitated by the continued development of robust and predictive animal models [229,233,310,311] and viral adaptation strategies [232] to quantify and qualify such responses.

## 7. Concluding Remarks

Over the past three decades, a multitude of studies have provided insights into the genetic organization and diversity, antigenicity and functional properties of the HCV envelope glycoproteins E1 and E2. However structural data concerning these fundamental viral proteins was limited. More recently, structural data for partial regions of both E1 and E2 have become available [42,49,71], in addition to partial E2 glycoprotein sequences in complex with multiple nAbs [62,63,65,66] revealing unusual structural attributes of these essential viral proteins. While these studies represent a significant advance, future studies should also aim to describe glycoprotein structures from diverse isolates and different stages of infection, for the purposes of comparison. In this review we demonstrate comparative analysis of current structural data reveals an additional and previously unappreciated layer of complexity on top of the observed genetic and antigenic heterogeneity: Structural flexibility in a conserved amino acid sequence when complexed with a different nAbs (see Figure 2). Additionally, we also mapped, at high-resolution, the amino acid diversity apparent in globally sampled genotype 1 viruses (the most prevalent genotype worldwide), onto the newly reported partial crystal structures for both E1 and E2 (see Figure 4). These analyses reveal the variable and conserved regions of both E1 and E2 together in a structural context for the first time.

Multiple functional studies indicate the glycoproteins E1 and E2 form heterodimeric complexes although structural information concerning the organization and orientation of these heterodimers is currently lacking and requires further investigation. Furthermore, it has been reported that E1 forms trimeric complexes [6,32] and mutations in E1 can increase the sensitivity of the E2 glycoprotein to neutralization with soluble CD81 [32] or E2 targeting mAbs [88], implying some re-organization of higher order structure which increases the accessibility of E2 epitopes. Whether repeating E1E2 heterodimers units form trimeric complexes and their molecular architecture remains unknown. Studies of HIV-1 virions have revealed unliganded and CD4 receptor-bound structures of the gp120 trimers, indicating a major re-organization of the trimer upon receptor binding [312], in addition to the distribution and availability of glycoprotein complexes over the surface of the virion [313]. Technological advancements and co-ordinated research efforts should facilitate analogous structural studies in HCV to elucidate the molecular architecture of glycoprotein complexes and their distribution over the virion surface. Indeed, E1E2s presented on the surface of authentic inactivated virions has been shown to be more immunogenic than the individual proteins expressed in isolation [288]. Structural information of intact glycoprotein complexes and the conformation changes induced by receptors binding, endosomal acidification and host lipoprotein association are additionally required. Current ultrastructural analyses of HCV virions revealed spherical particles between 40–100 nm in diameter with observable projections extending from the virion surface [50]. Interestingly, associated lipoproteins were more easily antibody-labelled than the viral glycoproteins, indicating either glycoprotein shielding by lipoproteins, low abundance on the virion surface, or both [50]. Thus, higher resolution studies are required to enable visualization of the lipoviral particle at high resolution and elucidate its precise molecular composition. In summary, an expansion of studies to address information gaps in our knowledge of glycoprotein structure should be guided by genetic, antigenic and functional studies to facilitate optimal immunogen design for use in HCV vaccine trials.
